# Cancer Risk in Autoimmune and Immune-Mediated Diseases: A Narrative Review for Practising Clinicians

**DOI:** 10.3390/jcm14175954

**Published:** 2025-08-23

**Authors:** David Bernal-Bello, Begoña Frutos-Pérez, Miguel Ángel Duarte-Millán, María Toledano-Macías, Beatriz Jaenes-Barrios, Alejandro Morales-Ortega

**Affiliations:** 1 Department of Internal Medicine, Hospital Universitario de Fuenlabrada, Fuenlabrada, 28942 Madrid, Spain; begona.frutos@salud.madrid.org (B.F.-P.); miguelangel.duarte@salud.madrid.org (M.Á.D.-M.); mtoledano@salud.madrid.org (M.T.-M.); alejandro.morales@salud.madrid.org (A.M.-O.); 2 Arroyomolinos Primary Health Care Center, Arroyomolinos, 28939 Madrid, Spain; beatriz.jaenes@salud.madrid.org

**Keywords:** autoimmune diseases, cancer risk, immune-mediated diseases, immunosuppressive therapy, clinical practice

## Abstract

**Background**: Autoimmune diseases and other immune-mediated disorders are associated with an increased risk of malignancy, influenced by chronic inflammation, immune dysregulation, and treatment-related factors. Clarifying cancer risk patterns across specific conditions is essential to improve clinical vigilance and inform screening practices. **Objective**: The aim of this study was to synthesise current evidence on the association between autoimmune and immune-mediated diseases and cancer, with a focus on practical implications for clinicians. **Methods**: Recent cohort studies, meta-analyses, and expert consensus documents were analysed to describe cancer epidemiology, pathogenic mechanisms, high-risk phenotypes, and treatment considerations across major autoimmune diseases and other immune-mediated conditions. The review covers idiopathic inflammatory myopathies, Sjögren’s syndrome, systemic sclerosis, systemic lupus erythematosus, rheumatoid arthritis, antiphospholipid syndrome, ANCA-associated vasculitis, giant cell arteritis, polymyalgia rheumatica, sarcoidosis, mixed connective tissue disease, IgG4-related disease, VEXAS syndrome, and eosinophilic fasciitis. Special attention was given to identifying warning features for underlying malignancy and evaluating cancer screening strategies. **Results**: The magnitude and distribution of cancer risk vary across diseases. In some conditions such as dermatomyositis, systemic sclerosis or Sjögren’s syndrome, increased risk is well established, particularly for haematological and certain solid tumours. However, tumour patterns may differ across populations, and findings are not always consistent. Distinct clinical and serological features help stratify individual cancer risk and may guide the intensity of screening. The first years after disease onset often represent a window of higher vulnerability, during which intensified surveillance may be warranted in selected patients. **Conclusions**: Cancer risk in autoimmune diseases should be assessed on an individual basis. Awareness of disease-specific risk factors and clinical warning signs supports early recognition of malignancy and informs screening decisions in routine practice.

## 1. Introduction

Autoimmune diseases (AIDs) are chronic disorders characterised by dysregulated immune responses against self-antigens [1,2,3]. In recent decades, the complex and bidirectional relationship between autoimmunity and malignancy has attracted growing attention as increasing evidence suggests shared mechanisms and overlapping pathways [4,5,6]. Certain AIDs, such as dermatomyositis and Sjögren’s syndrome, are well established as conferring a higher risk for specific cancers, particularly certain lymphomas and selected solid tumours [4,7,8].

**Figure 1:**
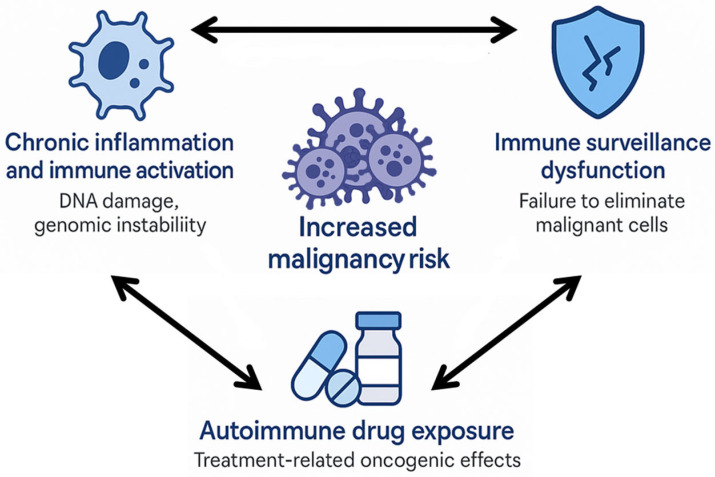
Pathogenic mechanisms linking autoimmunity and malignancy.

Several interconnected mechanisms may contribute to the bidirectional link between autoimmune diseases and malignancy (Figure 1). Persistent immune activation promotes chronic inflammation, oxidative stress, and DNA damage, driving genomic instability and tumour initiation [5]. Impaired immune surveillance—a hallmark of autoimmunity—facilitates malignant cell escape and unchecked proliferation [9,10]. Conversely, tumour antigens can induce autoimmunity through molecular mimicry, epitope spreading, or autoantibody responses against tumour-associated antigens [3,6,8], as exemplified in cancer-associated myositis and systemic sclerosis with anti-RNA polymerase III antibodies [1,2,8]. The use of immunosuppressive or immunomodulatory therapies adds further complexity. Alkylating agents have been linked to increased risks of haematologic and bladder cancers [7], while newer biologics such as TNF-α and JAK inhibitors have raised concerns about solid tumours and skin cancer in certain populations [5]. Distinguishing treatment-related risks from disease-related risks remains challenging and requires robust longitudinal studies.

This narrative review provides an up-to-date synthesis of the evidence linking AIDs and other immune-mediated systemic disorders with malignancy. We summarise the main epidemiological data, pathophysiological mechanisms and cancer patterns across major diseases, drawing on recent cohort studies, meta-analyses and expert consensus to inform cancer risk stratification and management in these patients. To this end, we performed a comprehensive, non-systematic literature search using PubMed/MEDLINE, Scopus, and Google Scholar. Particular attention was given to recent and clinically relevant publications, especially from the last decade, including large-scale studies, registry data, systematic reviews, and key original contributions. Additional references were identified through backward citation tracking and author expertise. No formal protocol or critical appraisal tool was applied, consistent with the narrative nature of this review.

## 2. Idiopathic Inflammatory Myopathies

- *Idiopathic inflammatory myopathies (IIMs) are autoimmune diseases with a well-established link to cancer, especially dermatomyositis, which shows the highest risk compared to other subtypes.*
- *Most cancer cases in IIMs appear within the first years after diagnosis, emphasising the importance of early vigilance and systematic screening.*
- *Recognising clinical predictors like age, sex, disease severity and key autoantibodies is crucial to stratify patients and guide the intensity of cancer surveillance.*
- *Evidence-based guidelines recommend adjusting screening to each patient’s risk profile, combining basic tests with advanced imaging when needed to improve early detection and prognosis.*

IIMs are a heterogeneous and rare group of chronic autoimmune diseases primarily characterised by muscle inflammation and frequent systemic involvement [11]. Major subtypes include dermatomyositis (DM), polymyositis (PM), overlap myositis (including antisynthetase syndrome, ASyS), immune-mediated necrotizing myopathy (IMNM), and inclusion body myositis (IBM).

Since the link between myositis and malignancy was first described [12], numerous studies have confirmed this association [13,14,15,16]. The risk appears to follow a specific temporal distribution, with the highest likelihood of cancer occurring from three years before to three years after IIM diagnosis [17,18], peaking within the first year. This temporal clustering supports the concept of cancer-associated myositis and may reflect shared mechanisms such as immune dysregulation, chronic inflammation, and the expression of tumour-associated antigens, particularly in DM [3,4,5,6]. Malignancy remains one of the leading causes of mortality in this group, as many cases are detected at advanced stages [19].

Not all IIM subtypes present the same degree of oncological risk [15,20]. DM has the strongest link, whereas PM is associated with a moderate increase. Amyopathic DM may confer a slightly higher probability than the general population. IMNM appears to carry an elevated chance of malignancy, mainly in individuals without myositis-specific antibodies or those with anti-HMGCR (3-hydroxy-3-methylglutaryl-coenzyme A reductase) positivity. In contrast, ASyS, juvenile DM and IBM are not clearly related to an excess risk.

The types of malignancy also vary by region. In Northern Europe, DM is most often linked to ovarian, lung, pancreatic, gastric and colorectal tumours as well as non-Hodgkin lymphoma, while PM is frequently associated with non-Hodgkin lymphoma, lung and bladder cancer [21]. In Southeast Asia, DM more often coincides with head and neck tumours, followed by lung and breast cancer, while PM is linked to colorectal, lung and hepatocellular carcinomas [22].

Several clinical, demographic and laboratory features help stratify this risk [15,16,20]. Relevant factors include age at diagnosis over 45, male sex, dysphagia—especially when severe—skin ulceration or necrosis, and refractory or aggressive disease. Conversely, interstitial lung disease, Raynaud’s phenomenon and arthritis are associated with a reduced malignancy risk. Markedly elevated creatine phosphokinase and lactate dehydrogenase levels may also suggest lower probability, whereas erythrocyte sedimentation rate shows no consistent link.

Among autoantibodies, anti-TIF1γ (transcription intermediary factor 1-gamma) is particularly relevant, as it targets proteins involved in oncogenesis [20,23]. Its association with malignancy can persist for up to ten years, justifying prolonged follow-up in anti-TIF1γ–positive individuals compared to those who are negative [18]. This association is not seen in juvenile dermatomyositis (DM) [23]. Anti-NXP2 (nuclear matrix protein 2) is also linked to increased malignancy risk in adults, but not in younger patients. A possible relationship has been suggested for anti-SAE1 (small ubiquitin-like modifier activating enzyme) antibodies, though supporting evidence is limited and a consistent temporal link has not been confirmed. In IMNM, anti-HMGCR antibodies may be associated with an elevated cancer risk, particularly in patients without coexisting antibodies.

In contrast, the presence of anti-Mi2 and anti-MDA-5 (melanoma differentiation-associated gene 5) antibodies does not appear to significantly increase malignancy risk [20]. Similarly, patients positive for antisynthetase antibodies (such as anti-Jo1, PL7, PL12, EJ, KS or ZI) or anti-SRP (signal recognition particle) seem to have a lower probability of associated cancer [20,23]. Interestingly, the co-expression of certain antibodies—particularly anti-CCAR1 (cell division cycle and apoptosis regulator 1) alongside anti-TIF1γ—may reduce cancer risk to levels comparable to the general population [24].

To improve early detection and reduce malignancy-related mortality, international groups such as IMACS (International Myositis Assessment and Clinical Studies Group) have developed evidence-based recommendations to identify IIM patients who may benefit from more intensive screening [23]. These recommendations stratify individuals according to the number and type of risk factors (Table 1): two or more high-risk factors indicate high risk; two or more intermediate factors or one high-risk factor indicate moderate risk; and patients without clear risk factors are assumed to have a probability similar to that of the general population.

Testing for myositis-specific autoantibodies is recommended for all newly diagnosed cases to ensure appropriate risk stratification. Screening should be adjusted accordingly. For individuals at standard risk, a basic assessment is advised including clinical history, physical examination, complete blood count, liver function tests, erythrocyte sedimentation rate, C-reactive protein, protein electrophoresis, urinalysis and chest radiograph. Those at higher risk should also undergo extended investigations including computed tomography (CT) scans of the neck, chest, abdomen and pelvis; cervical cytology; mammography; prostate-specific antigen; CA-125 testing; transvaginal ultrasound and faecal occult blood testing. In selected high-risk scenarios, positron emission tomography–computed tomography (PET-CT) may be warranted, especially for anti-TIF1γ-positive adults over 40 with additional factors. In areas with high incidence of nasopharyngeal carcinoma, nasoendoscopic assessment should be considered [23].

Clinicians should remain alert for warning signs such as unexplained weight loss, family history of malignancy, smoking, persistent fever or night sweats. All patients should continue to follow the recommended population-based screening guidelines in their region.

## 3. Sjögren’s Syndrome

- *Sjögren’s Syndrome (SS) is one of the autoimmune diseases most strongly associated with non-Hodgkin lymphoma (NHL), illustrating how chronic immune activation can lead to malignancy.*
- *The risk of lymphoma in SS increases progressively over time, which highlights the need for long-term vigilance.*
- *Parotid gland enlargement, lymphadenopathy, cryoglobulinaemia, low complement levels and monoclonal gammopathy are important clinical ‘red flags’ that help identify patients who may benefit from closer monitoring.*
- *Beyond lymphoma, SS has also been linked to an increased risk of certain solid tumours, although this association is less consistent and varies by tumour type.*

SS is characterised by lymphocytic infiltration of the exocrine glands, causing ocular and oral dryness. It may also produce extraglandular manifestations involving the lungs, nervous system or haematological system, among others [25]. For decades, SS has been known to carry an increased risk of malignancy, especially haematological neoplasms. A recent meta-analysis including over 47,000 patients estimated that the risk of developing haematological malignancies is 11.6-fold higher than in the general population [26]. Among these, NHL is the most common, with a relative risk estimated at 13–14 times higher in previous studies [27].

This SS–NHL link is stronger than that reported for other autoimmune diseases such as systemic lupus erythematosus (NHL risk seven times higher) or rheumatoid arthritis (four times) [28]. Approximately 5–10% of SS patients are estimated to develop NHL during the course of the disease [29,30]. The cumulative risk increases over time, with reported prevalence of 4% within the first five years, reaching up to 18% after 20 years [31]. The most frequent NHL subtype is low-grade B-cell lymphoma, particularly extranodal marginal zone mucosa-associated lymphoid tissue (MALT) lymphoma, typically affecting the salivary glands such as the parotid. Although these lymphomas are usually indolent, they may progress to more aggressive forms such as diffuse large B-cell lymphoma [32].

In addition to NHL, SS has also been linked to an increased risk of other haematological malignancies [26], with an estimated risk of being 8–9 times higher for Hodgkin lymphoma and multiple myeloma, and about three times higher for leukaemia.

Whether SS also increases the risk of solid tumours remains more controversial. Recent reviews suggest a consistently elevated risk, though much lower than for haematological neoplasms. The overall risk of solid tumours in SS is estimated at 1.5–2 times higher [26,27], mainly due to associations with certain specific cancers identified in various international cohorts. Notably, thyroid carcinoma has been reported with an incidence 2–5 times higher than expected [33,34]. Some studies indicate a 2–4-fold increased risk for oropharyngeal, gastric, lung or non-melanoma skin cancer [26,33]. In some cases, SS has been linked to urothelial, liver or prostate cancer, although data are more inconsistent [35]. The relationship with breast cancer remains controversial. While previous cohorts did not find a significant increase, some studies suggest geographical differences, with a lower risk in European populations and a possible higher risk in Asian populations [36,37].

SS is marked by persistent humoral immune activation, with ectopic germinal centres and lymphocytic infiltration of salivary and MALT. B-cell survival is sustained by key cytokines such as BAFF (B-cell activating factor, BLyS) and APRIL (A proliferation-inducing ligand), both overexpressed in SS. This microenvironment favours abnormal B-cell differentiation, and chronic lymphostimulation together with pro-oncogenic factors or loss of immune tolerance can drive neoplastic clone expansion [38,39]. Some viral triggers (e.g., Epstein–Barr virus, cytomegalovirus, other lymphotropic viruses) have also been studied as potential persistent antigenic stimuli maintaining BAFF production and favouring oncogenesis, although direct evidence remains limited [40,41].

Several studies have identified clinical and serological predictors that increase lymphoma risk in SS. Consistently reported factors include parotid enlargement and lymphadenopathy; presence of cryoglobulins and rheumatoid factor positivity; complement consumption (especially C4); and monoclonal gammopathy (Table 2). These markers are thought to reflect B-cell immune activation, predisposing to lymphomagenesis and this is exemplified by the predominance of low-grade B-cell lymphomas in the salivary glands [33]. Disease activity indices have also been shown to correlate positively with lymphoma risk [33,42].

The most widely used treatments for SS are glucocorticoids, classic steroid-sparing immunosuppressants, and rituximab [43]. Their influence has dual implications regarding malignancy risk: on one hand, possible increased cancer susceptibility due to effects on tumour immunosurveillance; on the other, a hypothetical reduction in the likelihood of neoplastic disease by controlling chronic lymphocyte stimulation, since active disease is more prone to lymphoma [44]. As for rituximab, studies in other immune-mediated conditions such as multiple sclerosis or rheumatoid arthritis do not suggest a higher oncological risk [45], and a specific SS series has found no excess incidence either [46].

## 4. Systemic Sclerosis

- *Systemic sclerosis SSc has a recognised increased risk of malignancy, especially lung and haematological cancers.*
- *A bimodal pattern of cancer occurrence has been described in SSc, with a peak in the early years after disease onset—supporting a possible paraneoplastic link—and a second increase later in the disease course.*
- *Specific autoantibodies (anti-RNA polymerase III) have been linked to higher malignancy risk, whereas others or negativity for anti-centromere/anti-topoisomerase I may help refine risk stratification.*
- *Current evidence highlights the need for tailored cancer screening, combining standard population-based tests with additional imaging or targeted work-up in high-risk patients.*

SSc is a chronic connective tissue disease characterised by fibrosis, vasculopathy and autoimmunity, which can affect multiple organs [47]. Its association with malignancy development has long been recognised, both as an intrinsic risk factor for certain tumours and through the paraneoplastic hypothesis, especially in well-defined clinical and serological contexts. Cancer is also one of the main causes of mortality in SSc patients [48].

Several mechanisms may underlie the increased malignancy risk in SSc. Chronic inflammation, persistent tissue damage and immune dysregulation create a pro-oncogenic microenvironment, particularly in fibrotic organs such as the lungs and gastrointestinal tract [8,48]. In patients with anti-RNA polymerase III antibodies (ARA), somatic mutations in the *POLR3A* gene within tumour tissue may lead to the expression of neoantigens that trigger both antitumour and autoimmune responses, supporting the concept of paraneoplastic autoimmunity in a subset of patients [2,6,48].

Robust epidemiological evidence [49,50,51,52], including large population-based meta-analyses [53,54,55], confirms an increased overall malignancy risk in SSc, consistently showing a higher risk compared to the general population. A bimodal pattern of cancer occurrence has been described [56,57]: the risk appears to be highest during the first years after SSc onset—supporting the idea that, in some cases, the disease may reflect an underlying paraneoplastic process [48,56,57,58]—and may increase again later in the disease course.

The highest and most consistent excess risks are observed for lung cancer—which remains the leading cause of cancer-related mortality in SSc—and haematological neoplasms, with standardised incidence ratios often two to four times higher than in the general population, depending on sex, cohort and time since diagnosis. For lung cancer, the risk is especially high in those with significant interstitial lung disease (ILD) and a history of smoking [49,59]. Regarding haematological malignancies—particularly NHL—variations in subtype definitions and sample sizes mean that precise estimates still vary [53,54,55,60]. In contrast, evidence for breast cancer is conflicting, with no clear excess in pooled data [53,54,55], although some individual series suggest a modestly increased risk [52,61].

A key aspect is the role of SSc-specific autoantibodies. Patients with ARAs have an increased risk of synchronous or closely related cancers, particularly of the breast and lung [56,62,63]. This distinctive temporal link reinforces the paraneoplastic hypothesis in this subgroup. By contrast, anti-centromere antibodies (ACAs) and anti-topoisomerase I antibodies (ATAs) do not show a consistent association with specific cancer types, although ATA positivity is clinically relevant due to its association with severe ILD, which may indirectly raise lung cancer risk [49]. A possible link between anti-PM/Scl antibodies and increased malignancy risk has been suggested [64,65], although other series have not confirmed a clear association [66]. Recent studies suggest that anti-PM/Scl positivity may also be related to non-melanoma skin cancers [57], but limited sample sizes prevent firm conclusions. Overall, the clinical value of anti-PM/Scl as a cancer risk marker remains to be clarified.

Recent evidence indicates that certain emerging autoantibodies may help stratify malignancysusceptibilityin SSc: anti-RNPC-3 (also known as anti-U11/U12) and anti-NVL (nuclear valosin-containing protein-like) have been linked to a higher likelihood of malignancy, particularly in cases with synchronous cancer [67,68] while anti-RPA194 (RNA polymerase I) and anti-Th/To may be associated with a reduced probability [69,70]. Additionally, cancer-associated SSc has been reported more frequently in patients negative for both ACA and ATA, particularly in diffuse disease, although triple negativity for ACA, ATA and ARA has recently been linked with increased breast cancer risk [71].

Although potential oncological hazards from immunosuppressive therapy are covered elsewhere, cyclophosphamide has been associated with an increased incidence of bladder cancer in SSc patients [72], but its relationship with other cancers is not clear or consistent [57,64,71]. A multicentre study observed that the use of calcium channel may correlate with a greater occurrence of overall malignancy, breast cancer and melanoma [73], while low-dose aspirin could have a protective effect against overall cancer according to a single-centre study [64].

There are no universally accepted recommendations for cancer screening in SSc beyond standard population-based strategies, but the literature emphasises the need for an individualised assessment based on clinical and serological risk factors [62,63], which should be intensified during the first years after diagnosis when risk is highest (Table 2).

Malignancy screening should at least include routine tests for the general population, such as mammography, cervical cytology, faecal occult blood testing and/or colonoscopy. In the absence of a universal strategy, screening decisions should be tailored to individual risk profiles. In higher-risk cases, additional imaging such as chest, abdominal and pelvic CT and targeted screening guided by symptoms or clinical findings may be considered [63,73,74]. PET/CT may also be considered if signs suggest occult malignancy. The routine use of serum tumour markers for screening is debatable and not justified. Intensive surveillance should be maintained for two to five years after diagnosis; after this period, systematic close oncological follow-up is not generally recommended [63].

## 5. Systemic Lupus Erythematosus

- *Systemic lupus erythematosus (SLE) is associated with a moderately increased malignancy risk, particularly lymphomas and Human Papillomavirus (HPV)-related cancers such as cervical, vulvar, and anal cancer.*
- *Certain cancers, including breast, prostate, and ovarian cancer, may occur less frequently in SLE, possibly due to hormonal or immunological factors.*
- *Cyclophosphamide exposure has been linked to a higher malignancy risk, while hydroxychloroquine may offer a protective effect.*
- *There are no disease-specific cancer screening guidelines for SLE, but annual cervical screening is recommended for women on immunosuppressive therapy.*

Multiple studies have shown that the incidence of malignant neoplasms in cohorts of patients with SLE is higher than would be expected compared to the general population or matched controls [75,76,77,78,79,80]. Most meta-analyses estimate that the increased risk of malignancy in patients with SLE ranges between 16% and 33% [81,82,83], although some have reported even higher figures, up to 44% [84] or 62% [85]. Some of these associations may reflect underlying mechanisms such as sustained immune activation, altered hormonal milieu, or differential exposure to immunosuppressive therapy, although causality remains difficult to prove, and likely varies between cancer types [3,4,5,6].

The strongest and most consistent association is with haematological malignancies, particularly NHL, which occurs up to 4–5 times more frequently in SLE patients than in the general population. Elevated risks have also been described for Hodgkin lymphoma, leukaemia, myelodysplastic syndromes and, more controversially, multiple myeloma [79,80,83,85,86,87,88,89,90,91]. The next strongest association links SLE to cancers of the cervix, vulva and vagina, occurring 3–4 four times more frequently in these patients [76,78,82,83,85,91,92]. This may be explained by a higher susceptibility to persistent HPV infection and reduced viral clearance due to immunosuppressive treatment [92]. In line with this, recent meta-analyses have also confirmed an increased risk of anal cancer in SLE [83,85,93].

Associations with other solid tumours are generally weaker and more variable. Recent single-centre cohort data have reported a malignancy prevalence of around 10% in SLE, with non-haematological neoplasms predominating—particularly in patients with older age at onset and longer disease duration [94]. Lung cancer has been reported more frequently in SLE cohorts [80,82,83,85,91,95], as have cancers located in the neck (pharynx, larynx or thyroid) [76,80,82,83,85,91,96,97] and hepatobiliary system, particularly hepatocellular carcinoma [75,80,82,83,85,91,98]. Several studies have suggested an increased risk of bladder cancer in SLE patients [78,85,91], although conflicting data exist [80]. Cyclophosphamide exposure has been proposed as a potential driver of bladder cancer risk, though definitive evidence is still lacking. The evidence for renal cancer is similarly conflicting [83,85,91] and the relationship between SLE and gastrointestinal malignancies remains controversial: while some studies report higher risks of oesophageal [85,91], gastric [83,91], colorectal [83,85] and pancreatic cancers [80,85,99], others have not confirmed these findings [83,85,91].

By contrast, most studies agree that SLE is not associated with an increased risk of ovarian cancer [83,85,91]. Similar findings have been reported for breast, prostate and endometrial tumours, and some studies have even suggested a decreased risk in patients with SLE [75,81,83,91,100]. This reduction might be partly explained by hormonal factors, as SLE is associated with later menarche and earlier menopause in women [101]. Other hypotheses include a potential protective effect of antimalarial drugs, or that certain autoantibodies may suppress tumour progression by affecting molecules such as Hsp27, or directly interfere with DNA repair processes in tumour cells already compromised in this regard [92,101,102,103,104].

Finally, an increased risk of non-melanoma skin cancer has been reported in SLE patients, while the risk of melanoma appears lower [83,85,91]. This paradox may be due to intentional sun avoidance in these patients [101] or increased AIM2 (Absent in Melanoma 2) protein levels, which have tumour-suppressive effects in cancers such as melanoma, breast and prostate, but may increase risk in others like lung cancer [105]. However, these mechanisms remain speculative and require further research.

Beyond general immune dysregulation common to many autoimmune diseases, additional mechanisms have been proposed to explain the link between SLE and malignancy, including increased synthesis of the APRIL protein (which may promote apoptosis evasion and has been observed in diffuse large B-cell lymphomas in SLE), shared genetic predispositions involving tumour necrosis factor (TNF) and interferon pathways, possible overlap with secondary SS and the impact of immunosuppressive therapies [101]. In this sense, certain commonly used treatments have shown varying associations with malignancy risk, although evidence remains conflicting [106]. Cyclophosphamide has been linked to a higher risk of malignancies—especially haematological neoplasms, non-melanoma skin cancers or cervical dysplasia and cervical cancer—with a dose-dependent relationship [107,108]. Antimalarial drugs, particularly hydroxychloroquine, have been associated with a lower overall malignancy risk in SLE. This protective effect appears dose-dependent and has been described for overall, breast cancer and non-melanoma skin tumours [108,109,110,111]. No clear link has been found between other immunosuppressants (such as azathioprine, methotrexate or mycophenolate mofetil) or glucocorticoids and an increased overall malignancy risk, although some data suggest a possible higher risk of lymphoma with high cumulative steroid doses and long-term immunosuppressant exposure [107,108].

There are no specific cancer screening guidelines for SLE beyond those recommended for the general population. However, enhanced cervical cancer surveillance with annual Pap smears are advised, especially for women receiving immunosuppressive treatments such as cyclophosphamide [112]. For patients previously exposed to cyclophosphamide, some experts also suggest considering urine cytology to screen for bladder cancer, though not universally endorsed [113].

## 6. Rheumatoid Arthritis

- *Rheumatoid arthritis (RA) is associated with a modestly increased overall cancer risk, especially for lung cancer and lymphomas, while colorectal and breast cancer risks may be slightly reduced.*
- *Risk factors for malignancy in RA include smoking, high disease activity, ILD, seropositivity, and extra-articular features such as Felty’s syndrome.*
- *Effective inflammation control reduces cancer risk; methotrexate and TNF inhibitors do not significantly increase overall malignancy rates, though Janus Kinase (JAK) inhibitors require caution in high-risk patients.*
- *Cancer screening in RA should follow general population guidelines, with particular attention to cervical and skin cancer surveillance in immunosuppressed individuals.*

Patients with RA have a moderately increased overall malignancy risk compared to the general population, estimated to be around 10% higher [114]. The most consistently elevated risks are seen for lung cancer and haematological malignancies, particularly B-cell and T/NK-cell NHL [115,116,117]. The risk of lung cancer in RA is approximately 1.5 to 3.5 times higher than in individuals without RA, partly explained by smoking and coexisting ILD [118]. Large granular lymphocyte (LGL) leukaemia, a rare clonal disorder, is also strongly associated with RA, especially in patients with Felty’s syndrome [119]. LGL clonal expansions may emerge after long-standing RA or even precede diagnosis, reflecting shared immune mechanisms. Additionally, there is evidence of an increased incidence of acute myeloid leukaemias and myelodysplastic syndromes in patients with RA, particularly in seronegative forms of the disease [120].

In contrast, several studies and meta-analyses have reported a slightly reduced risk of colorectal and breast cancer in RA, possibly related to prolonged non-steroidal anti-inflammatory drugs (NSAID) use and chronic anti-inflammatory treatment [114,121]. The reduced breast cancer incidence has been attributed to hormonal factors, genetic polymorphisms, and interactions within the tumour microenvironment [122]. The strongest evidence for the reduced risk of colorectal cancer highlights the protective role of NSAIDs and cyclooxygenase-2 inhibitors [123]. There may be a slight increase in cervical dysplasia and cervical cancer risk in women with RA treated with immunosuppressive agents [124], although the evidence is inconclusive and the absolute risk is considered low.

Known risk factors for malignancy in RA include smoking, seropositivity for rheumatoid factor or anti-citrullinated peptide antibodies (ACPA), older age, male sex, features shared with Sjögren’s syndrome, and extra-articular manifestations such as ILD or Felty’s syndrome (Table 2) [114,123,125,126]. High disease activity and persistent inflammation have also been identified as key drivers of oncogenesis, particularly for haematological malignancies, and may play a more prominent role than genetic predisposition [127].

Despite the potential risks of some treatments, effective RA therapy that suppresses chronic inflammation is generally considered to lower the overall risk of malignancy [117]. Methotrexate (MTX) was initially linked to rare lymphoproliferative disorders, often associated with Epstein–Barr virus, some of which resolved spontaneously after MTX discontinuation [128,129]. Similarly, indirect evidence suggests that this drug may increase the incidence of non-melanoma skin cancer [130]. However, large cohort studies have not demonstrated a significant increase in overall solid tumour risk attributable to MTX [131]. Unlike in SLE, there is no evidence that antimalarial drugs, such as hydroxychloroquine, confer any protective effect against cancer in RA.

TNF inhibitor (TNFi) safety in relation to cancer risk has been extensively studied. Initial concerns about a possible dose-dependent increase in lymphoma risk with TNFi use [132] have not been confirmed in subsequent large-scale analyses, which show no significant excess risk compared to conventional disease-modifying anti-rheumatic drugs [116,133]. Some data suggest a modestly increased risk of follicular lymphoma, non-melanoma skin cancer, and high-grade cervical dysplasia or cervical cancer associated with TNFi therapy [124,134].

JAK inhibitors, such as tofacitinib, have raised safety concerns, particularly after the ORAL Surveillance triall [135], which reported higher rates of overall malignancy—including lung cancer and lymphoma—compared to TNFi, particularly in older patients with cardiovascular risk factors. Consequently, current recommendations advise reserving JAK inhibitors for patients who have failed TNFi therapy and who do not have high baseline cancer risk [136].

There are no specific cancer screening guidelines for RA beyond the general population recommendations. Therefore, monitoring aligned with age-based guidelines, vigilance for red flags or skin changes, and encouraging adherence to cervical cancer screening programmes remain essential for the early detection of malignancy in patients with RA [114,118,124].

## 7. Antiphospholipid Syndrome

- *Antiphospholipid syndrome (APS) has been associated with malignancy, particularly in patients with unexplained thrombosis or refractory disease.*
- *Antiphospholipid antibodies positivity is common in patients with solid tumours—especially gastrointestinal, genitourinary, and lung cancers—and is linked to increased thrombotic risk.*
- *Catastrophic APS may occur in the context of cancer, often triggered by the malignancy or related interventions like surgery.*
- *While APS may occasionally be paraneoplastic, routine cancer screening beyond standard population guidelines is not currently recommended.*

The link between unexplained thrombosis and underlying malignancy has long been recognised, and multiple studies have explored a possible association between APS and cancer development [137,138]. The Euro-Phospholipid Project Group reported that cancer was the second leading cause of death among APS patients in their large international cohort [139]. Isolated cases of antiphospholipid antibodies (aPL)—with or without overt thrombosis—have been described in patients with cancer [137].

A systematic review of 33 studies found a high prevalence of aPL (anticardiolipin, lupus anticoagulant, and anti-β2 glycoprotein I) in patients with solid tumours, especially gastrointestinal, genitourinary, and lung cancers, and confirmed the link with increased thrombotic risk [140], possibly reflecting shared mechanisms such as chronic inflammation, endothelial dysfunction, and a prothrombotic microenvironment. Some local cohorts have reported anticardiolipin as the most common aPL in cancer patients [141,142], while others have found higher rates of anti-β2 GPI IgA [143] or lupus anticoagulant positivity [144].

Cases of catastrophic APS (CAPS) have also been described in oncology settings. The international CAPS registry found that about 9% of cases were cancer-associated, with haematological malignancies being the most frequent, followed by lung and colon cancer. Surgery or the underlying tumour itself often act as triggers for CAPS in these patients [145]. Furthermore, an increased risk of malignancy has been observed in women with pregnancy losses associated with obstetric APS [137].

Given this overlapping evidence, some authors have proposed that APS might occasionally represent a paraneoplastic syndrome. Others suggest that malignancy can precede APS development or that aPL titres may decrease after successful cancer treatment, highlighting the complex interplay between the prothrombotic state of malignancy and APS onset [146]. Chronic hypercoagulability may itself promote tumour growth [147], and some studies have discussed a potential dual role of aPL in tumour biology and thrombosis [142].

Taken together, current evidence supports a clinically meaningful association between APS and malignancy, and hidden neoplasia should be considered in APS patients who have an unfavourable course despite appropriate treatment. However, routine systematic cancer screening is not recommended in patients with antiphospholipid syndrome beyond standard population-based protocols according to age, sex, and individual risk factors.

## 8. ANCA-Associated Vasculitis

- *Patients with ANCA-Associated Vasculitis (AAV) have an increased malignancy risk, historically driven by cyclophosphamide exposure.*
- *Squamous cell carcinomas—most notably those of the lung—are emerging as the most common malignancies, possibly related to chronic inflammation and underlying immune dysregulation.*
- *Cyclophosphamide remains a key risk factor, with dose-dependent associations, while rituximab appears to have a safer oncologic profile.*
- *Cancer screening should follow general population guidelines, with focused skin surveillance in those exposed to immunosuppressants.*

Recent studies exploring the relationship between AAV and malignancy have consistently reported an increased risk of malignancy in this population. At least four national cohorts, each comprising several hundred patients, have shown cancer incidence rates two to three times higher than in the general population [148,149,150,151]. The largest of these, including over 500 patients of Asian origin, identified lung cancer as the most frequently diagnosed malignancy in AAV, followed by urothelial (renal, bladder, ureteral), prostate, pancreatic, and non-melanoma skin cancers [148,150,151]. Overall, squamous cell carcinoma –particularly in the lung- appears to be the most common histological subtype, with a significantly increased frequency [150]. Historically, bladder cancer, certain myeloid leukemias, and non-melanoma skin cancer were the most common malignancies, largely driven by cyclophosphamide use. However, their incidence has decreased in more recent series [152]. These evolving patterns suggest that malignancy in AAV may not only reflect treatment-related risks but also shared pathogenic mechanisms such as sustained inflammation and impaired immune surveillance.

Risk factor analyses have identified male sex, older age, and anti-proteinase-3 positivity (compared to anti-myeloperoxidase) and cyclophosphamide exposure as potential contributors to malignancy development (Table 2) [148,149]. By disease subtype, granulomatosis with polyangiitis (GPA) shows the highest overall risk, particularly for haematological, bladder and lung neoplasms. In microscopic polyangiitis (MPA), the excess is mainly related to lung cancer, whereas in eosinophilic granulomatosis with polyangiitis (EGPA) it is more strongly linked to haematological malignancies [148,149,150].

Some authors have proposed shared immunological mechanism between AAV and cancer As noted, AAV onset appears to increase the likelihood of subsequent malignancy. A high proportion of patients develop bronchial or pulmonary inflammatory lesions, and chronic inflammation is a known driver of carcinogenesis, particularly in lung cancer [153].

Additional pathways have been expored. Studies have reported high CTLA-4 (cytotoxic T-lymphocyte–associated protein 4) expression and low CD28 expression in AAV patients, markers associated with T-cell immunosenescence, that may impair tumour cell immune surveillance. Altered cytokine profiles, including increased IL-6, IL-10 and PD-L1, which participate in tumour progression, have also been documented [153,154].

Conversely, some authors suggest that, in a subset of cases, AAV could be triggered by a preceding or underlying malignancy. This hypothesis is supported by earlier reports describing a 4.8–10% cancer prevalence preceding or coinciding with AAV diagnosis [155,156,157]. However, more recent studies have not confirmed a significant excess before disease onset [158], suggesting that malignancy is more likely a consequence of chronic inflammation or immunosuppressive therapy than a causal trigger.

Prolonged exposure to cyclophosphamide—still recommended for severe forms of AAV [159]—has been directly linked to increased risk of malignancy, particularly bladder cancer, some forms of leukaemia, and non-melanoma skin cancers [152]. The risk rises with cumulative doses and treatment durations [151]. One study found an eleven-fold increase in cancer incidence among patients receiving more than 20 g of cumulative cyclophosphamide [148]. While some authors have reported an overall decrease in malignancy rates in more recent AAV cohorts—likely due to reduced use of cyclophosphamide—persistent rates of non-melanoma skin cancer have been partly attributed to the continued use of other immunosuppressants such as azathioprine [158]. Nevertheless, studies also show that patients with short disease duration or limited exposure to cyclophosphamide (<10 g) still exhibit a heightened risk of cancer, albeit to a lesser degree. These observations support a possible intrinsic oncogenic role of AAV itself [148,150].

Rituximab has garnered particular interest in this context. Now recommended for both the induction and maintenance of AAV [159], this agent appears to have a more favourable oncological profile. In a Turkish cohort, rituximab use was associated with a significantly lower risk of malignancy [149]. A key comparative study reported that cancer incidence in patients treated with cyclophosphamide was approximately three times higher than in the general population, whereas no increased risk was seen in the rituximab group. The relative risk of developing malignancy was estimated to be four times higher with cyclophosphamide than with rituximab [160].

In summary, patients with AAV have an increased malignancy risk, historically linked to cyclophosphamide exposure and the development of bladder, haematological and non-melanoma skin cancers. While the use of this agent has declined, emerging data suggest a potential rise in squamous cell carcinomas—particularly lung cancer—possibly driven by chronic inflammation and immune dysregulation. Future research will help refine risk stratification and clarify the long-term safety of different therapeutic strategies. Cancer screening in patients with AAV should follow standard population-based recommendations, with particular attention to skin surveillance in those previously treated with conventional immunosuppressants.

## 9. Giant Cell Arteritis and Polymyalgia Rheumatica

- *Patients with giant cell arteritis GCA or polymyalgia rheumatica (PMR) have a slightly increased overall malignancy risk, especially within the first year after diagnosis.*
- *Cutaneous, prostate, lung, kidney, and haematological malignancies are the most frequently reported cancers.*
- *Atypical clinical presentations may represent paraneoplastic syndromes, highlighting the need for careful differential diagnosis.*
- *Routine cancer screening beyond standard population-based protocols is not recommended, but targeted vigilance is warranted in high-risk cases.*

GCA and PMR are inflammatory conditions that predominantly affect older adults. Both disorders share immunopathogenic mechanisms, such as chronic systemic inflammation [161,162], which may contribute to an increased susceptibility to malignancy. Persistent immune activation, age-related clonal haematopoiesis and a proinflammatory milieu may act as facilitators of oncogenesis in this context. However, the magnitude and nature of this risk remain incompletely defined.

Population-based studies and meta-analyses [163,164,165,166,167] have shown a slightly increased overall malignancy risk in patients with GCA or PMR compared to the general population, with an estimated excess of around 15%. This elevated risk appears to be concentrated mainly in the first year following diagnosis, a period likely influenced by detection bias, co-diagnosis, or previously undiagnosed malignancies, rather than a direct causal relationship.

The most frequently reported cancers in this context include cutaneous neoplasms (both melanoma and non-melanoma), as well as lung, prostate, kidney, and haematological malignancies such as lymphomas, leukaemias, and multiple myeloma [164,165]. When excluding cancers diagnosed within the first year, a modest residual risk persists for certain tumour types—particularly melanoma, prostate cancer, and selected haematologic malignancies.

Male sex has been specifically identified as a risk factor for haematologic neoplasms, particularly of myeloid origin [165]. Other independent risk factors for malignancy in this population include older age at diagnosis, tobacco use, and medium-to-high socioeconomic status, the latter being more clearly associated with an increased risk of solid tumours (Table 2) [168].

The relationship between GCA/PMR and malignancy remains insufficiently characterised. Some authors have suggested that clonal haematopoiesis—a common phenomenon in the ageing population—may play a role in the shared pathogenesis of GCA and haematologic malignancies [165].

Importantly, several studies have emphasised the need to differentiate true PMR from paraneoplastic syndromes, particularly in older patients with atypical clinical features. In such cases, presentations mimicking new-onset PMR may in fact reflect occult cancer manifesting with inflammatory musculoskeletal symptoms [169].

To date, no laboratory markers have been identified to reliably predict malignancy risk in GCA/PMR, and the available evidence does not support routine use of PET/CT to screen for cancer in all patients with suspected disease [170]. Nonetheless, some authors advocate for fast-track referral pathways from primary care for patients with unusual clinical signs -such as unexplained weight loss, absence of prolonged morning stiffness-, poor response to corticosteroids or imaging findings not suggestive of PMR [171].

Currently, no specific cancer screening recommendations exist for patients with GCA or PMR beyond standard population-based guidelines tailored to age, sex, and individual risk factors. As such, appropriate clinical vigilance and personalised risk assessment are essential, particularly when the disease course is atypical or refractory to treatment.

## 10. Sarcoidosis

- *Sarcoidosis seems associated with an increased overall malignancy risk, particularly for lymphomas.*
- *Diagnostic challenges include sarcoid-like reactions near tumours or induced by immunotherapy.*
- *The link with solid tumours, including lung cancer, is inconsistent and may reflect detection bias.*
- *Clinical vigilance for haematological malignancies might be appropriate, depending on individual patient characteristics*

The association between sarcoidosis and an increased risk of cancer was first proposed by Brincker and Wilbek, who described a higher-than-expected incidence of haematological malignancies (up to 11-fold) and lung cancer (3-fold) in a Danish cohort of patients with this disease [172]. Given this marked link with haematological neoplasms, Brincker later coined the term sarcoidosis–lymphoma syndrome to describe patients who developed lymphoma, particularly Hodgkin’s disease, after a diagnosis of sarcoidosis [173]. The link between sarcoidosis and malignancy may reflect shared genetic or environmental factors that predispose to immune dysregulation. Chronic inflammation and impaired tumour surveillance may contribute to carcinogenesis, while cancer-related immune dysfunction may also promote granuloma formation and sarcoid-like reactions [174].

However, these early hypotheses were often based on isolated case reports or small series. Over time, the link between sarcoidosis and malignancy has become more debated, as larger controlled studies have yielded mixed or contradictory results [175,176,177,178]. Several factors complicate the interpretation of these findings: first, sarcoidosis patients often undergo extensive imaging at diagnosis, increasing the likelihood of incidental tumour detection [174]. Second, sarcoid-like reactions in lymph nodes or tissues adjacent to tumours —especially lymphomas and epithelial cancers—can lead to misdiagnosis of sarcoidosis if the underlying malignancy is still occult [179]. Third, sarcoid-like reactions can also be drug-induced, particularly by immune checkpoint inhibitors used in oncology [180].

Overall, current evidence suggests an increased malignancy risk in sarcoidosis. A 2015 meta-analysis of 16 controlled studies estimated this risk to be about 19% higher than in controls [181], and a recent large cohort study involving nearly 4000 patients reported an even greater increase of about 60% [178]. This excess risk is mainly related to haematological neoplasms, especially lymphomas. The estimated risk of lymphoma in sarcoidosis is 2–3 times higher than in the general population, although whether Hodgkin’s or non-Hodgkin’s subtypes predominate varies between studies [178,181].

By contrast, the link with non-haematological tumours remains less clear. Some studies report increased risks of skin (mainly non-melanoma), renal, or hepatic cancers [178,181,182], but findings are inconsistent, and the association with lung cancer is particularly debated. While some cohorts have shown an excess risk shortly after sarcoidosis diagnosis—likely due to detection bias—long-term data do not confirm a sustained increase [178,181,182]. Interestingly, some studies even suggest a slightly lower long-term lung cancer risk, possibly related to the inverse association between sarcoidosis and smoking seen in several reports [178,181].

In practice, clinicians should be alert to diagnostic pitfalls like sarcoid-like reactions when evaluating new lymphadenopathy or lesions in sarcoidosis patients. Although the absolute malignancy risk increase seems modest, targeted vigilance for haematological malignancy may be justified in selected high-risk individuals.

## 11. Other Conditions

***Mixed Connective Tissue Disease***

- *Mixed connective tissue disease (MCTD) has not been clearly associated with an overall increased malignancy risk, although isolated cases of lymphoma and solid tumours have been reported.*
- *Chronic immune activation and immunosuppressive therapy may contribute to oncogenesis; atypical or refractory cases should prompt malignancy work-up.*

***IgG4-Related Disease***

- *IgG4-Related Disease (IgG4-RD) is associated with increased risks of lung, pancreatic, and lymphoid cancers, particularly within three years of diagnosis.*
- *Some forms of IgG4-RD may represent immune paraneoplastic syndromes; cancer screening at diagnosis is considered reasonable.*

***VEXAS Syndrome***

- *VEXAS is strongly linked to haematological malignancies, with up to 55% of patients developing myeloid cancers.*
- *UBA1 mutations drive clonal haematopoiesis and inflammation, requiring regular monitoring for haematologic cancer.*

***Eosinophilic Fasciitis***

- *Eosinophilic Fasciitis (EF) may precede the diagnosis of lymphoproliferative neoplasms, particularly in older adults.*
- *Although no biomarkers predict malignancy risk, eosinophilia or atypical features should prompt evaluation for hidden cancer.*

**Mixed Connective Tissue Disease**. MCTD is a rare overlap syndrome that combines features of SLE, SSc, and IIMs, and is typically characterised by high-titer anti-U1 RNP antibodies [183,184]. Although MCTD shares clinical and immunological features with other connective tissue diseases associated with increased malignancy risk, robust population-based evidence remains limited, and no clear overall increase in cancer incidence has been demonstrated in patients with MCTD compared to the general population. Isolated case reports have described associations between MCTD and malignancies such as NHL, thymoma, ovarian cancer, papillary thyroid carcinoma, and hepatocellular carcinoma [185,186,187,188]. These observations raise the possibility of coincidental occurrence or under-recognised paraneoplastic mechanisms. Chronic immune activation, B-cell hyperactivity, and sustained interferon pathway upregulation may contribute to a pro-oncogenic environment, in line with findings in SS or SLE [38,41,92,100,101,102,103]. In selected severe cases, the use of immunosuppressive agents such as cyclophosphamide or azathioprine could also influence cancer risk, although direct data remain scarce. Overall, current evidence does not support intensified cancer screening beyond standard age- and sex-appropriate recommendations. However, clinicians should remain alert to atypical presentations or treatment-refractory disease that may warrant targeted evaluation for occult malignancy.

**IgG4-Related Disease**. IgG4-RD is characterised by fibroinflammatory lesions that can affect virtually any organ. Its diagnosis relies on clinical, serological, radiological and histopathological findings [189]. Because of its broad spectrum, IgG4-RD is often called a “great mimicker”, as it can easily be confused with solid or haematological malignancies, especially on imaging [190].

For years, the potential link between IgG4-RD and cancer has been debated, either as a risk factor or a paraneoplastic phenomenon. Several studies suggest an increased risk of lung, pancreatic, gastric, biliary, lymphoid and prostate cancers in patients with IgG4-RD [191,192]. Risk may vary by organ involvement: pancreatic manifestations, for instance, have been proposed as an independent risk factor for malignancy in multiple cohorts [190,193,194]. In autoimmune pancreatitis (AIP), increased rates of gastric and colorectal cancer are reported, mainly within the first year after diagnosis, while pancreatic cancer itself is less common than expected [195].

A 2022 systematic review and meta-analysis pooling 13 studies and over 4500 patients confirmed increased risks for pancreatic, lung and lymphoid neoplasms, particularly within three years of IgG4-RD diagnosis [196]. The pathogenesis remains unclear but may involve shared pathways, such as K-ras mutations found in gastrointestinal tissue of patients with AIP [197].

Some authors hypothesise that IgG4-RD might act as an immune paraneoplastic syndrome, supported by findings of IgG4-positive plasma cells within tumour stroma and reports of IgG4-RD remission after successful cancer therapy [195,198]. Despite some contradictory results—often due to small samples and heterogeneous designs—an increased malignancy incidence appears plausible [193,197,199]. Therefore, routine cancer screening at diagnosis may be reasonable in clinical practice, with active surveillance considered especially in patients with pancreatic involvement or other concerning clinical features.

**VEXAS Syndrome**. VEXAS syndrome (vacuoles, E1 enzyme, X-linked, autoinflammatory, somatic) results from somatic mutations in the UBA1 (ubiquitin-like modifier-activating enzyme 1) gene and is considered a prototype of acquired haematoinflammatory disease [200]. Unlike other autoinflammatory disorders, VEXAS is strongly associated with haematological malignancies, with 25–55% of patients developing leukaemia, multiple myeloma or lymphoma [201,202,203,204]. This risk exceeds that of other acquired bone marrow failure syndromes like paroxysmal nocturnal haemoglobinuria [205].

The UBA1 mutation disrupts protein ubiquitination, driving chronic inflammation and clonal expansion of abnormal haematopoietic cells [200]. Consequently, rigorous monitoring for haematologic cancer is essential in affected individuals (Table 2).

**Eosinophilic Fasciitis** is a rare fibrosing disease first described by Shulman in 1974 [206], characterised by skin induration, eosinophilia, and fascial inflammation [207]. Traditionally considered benign, more recent reviews suggest possible associations with underlying malignancy, with haematological malignancies being most common, particularly among older adults [208,209,210,211,212,213,214]. The strongest links have been reported with lymphoproliferative neoplasms, and EF may precede the cancer diagnosis. Solid tumours are considered uncommon, although isolated reports have described associations with breast cancer, colon cancer, and melanoma [215,216,217].

No specific clinical or laboratory markers have been identified to predict malignancy risk, but marked eosinophilia or atypical systemic features may raise suspicion and warrant investigation for an underlying neoplasm, especially in refractory cases or those with an unusual clinical course.

## 12. Practical Summary and Clinical Implications

The relationship between AIDs and other immune-mediated disorders and cancer is complex, multifactorial, and highly variable across conditions. While the overall cancer risk in many AIDs is only moderately increased, certain conditions—such as DM, SSc, and SS—display well-documented associations with specific malignancies, including lymphomas, lung cancer, and gynaecological tumours. In some cases, autoimmunity may act as a paraneoplastic phenomenon, while in others, persistent inflammation, immune dysregulation, and immunosuppressive therapies may promote the emergence of malignancy.

Given the heterogeneity of cancer risk across AIDs and immune-mediated disorders, no universal screening strategy is currently recommended. Instead, screening decisions should be tailored to individual risk factors, including age, sex, serological profile, disease subtype and severity, and treatment history. In general, all patients should adhere to population-based screening guidelines appropriate for age and sex, while selected high-risk individuals may benefit from additional imaging or targeted investigations.

To facilitate early detection of malignancy and improve patient outcomes, clinicians should carry out the following:

- Maintain heightened oncological vigilance during the first years following diagnosis, especially in conditions with a strong temporal cancer link (e.g., cancer-associated myositis or SSc with anti-ARA).
- Recognise key clinical and serological features that may warrant intensified cancer work-up (Table 2).
- Implement stratified screening protocols, adjusting intensity and modalities based on each patient’s risk profile (Table 3).
- Be cautious when interpreting new or atypical symptoms, particularly in patients with refractory disease, poor treatment response, or longstanding inflammation.

Finally, while the risk of malignancy associated with immunosuppressive treatments remains a concern, effective disease control is often more protective than harmful. Treatment choices should be balanced with cancer risk assessment and clinical context, especially in patients with prior neoplasia or strong cancer predisposition.

## Figures and Tables

**Table 1:** Malignancy risk factors and stratification in patients with idiopathic inflammatory myopathies (adapted from Oldroyd et al. [23]).

High Risk	Intermediate Risk	Low Risk
DM Age over 40 at diagnosis Treatment refractoriness or relapses Dysphagia (moderate/severe) Cutaneous necrosis or ulceration TIF1γ NXP2	Amyopathic DM Polymyositis Immune-mediated necrotizing myopathy Male sex SAE1 HMGCR Mi-2 MDA-5	ASyS Overlap syndromes Raynaud’s phenomenon Arthritis Interstitial lung disease Anti-SRP Anti-Jo1 Non–Jo-1 ASyS Anti–PM-Scl, Ku, anti-RNP, anti-Ro/La

DM, dermatomyositis; TIF1γ, transcriptional intermediary factor 1γ; NXP2, nuclear matrix protein 2; SAE1, small ubiquitin-like modifier-1 activating enzyme; HMGCR, 3-hydroxy 3-methylutaryl coenzyme A reductase; MDA-5, melanoma differentiation-associated gene 5; ASyS, anti-synthetase syndrome; SRP, signal recognition particle.

**Table 2:** Features suggestive of underlying malignancy.

Disease	Age, Sex	Clinical Findings	Serum Findings
IIMs *	>40 at diagnosis Male	Dysphagia Skin ulceration or necrosis Refractory or aggressive disease	TIF1γ, NXP2
Sjögren’s Syndrome		Persistent parotid enlargement Lymphadenopathy	Low C4 complement levels Monoclonal gammopathy Cryoglobulins, rheumatoid factor
Systemic Sclerosis	Onset > 65	Aggressive, atypical, or treatment-refractory disease course. Significant weight loss or other constitutional symptoms (disproportionate to the severity of the autoimmune disease). ILD (for lung cancer).	ARA Negative for ACA and ATA in SSc-diffuse disease
SLE		Significant weight loss or other constitutional symptoms (disproportionate to the severity of the autoimmune disease). Persistent HPV infection Prior cyclophosphamide exposure or long-term immunosuppression	
Rheumatoid Arthritis	Older age Male	Persistent inflammation or high disease activity ILD (for lung cancer) Felty’s syndrome (for large granular lymphocyte leukaemia)	Seropositivity
Antiphospholipid Syndrome		Unexplained or recurrent thrombosis despite therapy Refractory disease	
AAV	Older age Male	High cumulative exposure to cyclophosphamide	Anti-PR3 positivity
GCA/PMR	Older age Male	Atypical (e.g., absence of morning stiffness) or disproportionate symptoms Poor response to steroids	
Sarcoidosis		Disproportionate symptoms, atypical clinical or radiological course Refractory disease	
MCTD		Refractory or atypical clinical course Treatment-refractory disease	
IgG4-RD		Pancreatobiliary involvement Multi-organ disease High inflammatory activity	
VEXAS Syndrome		Bone marrow dysplasia at diagnosis.	Persistent cytopenias Somatic mutation UBA1
Eosinophilic Fasciitis		Atypical systemic features Refractory or unusual clinical course	Marked eosinophilia

* See also Table 1. IIMs, idiopathic inflammatory myopathies; TIF1γ, transcriptional intermediary factor 1γ; NXP2, nuclear matrix protein 2; ILD, interstitial lung disease; ARA, anti-RNA polymerase III; ACA, anti-centromere antibodies; ATA, anti-topoisomerase I; SLE, systemic lupus erythematosus; HPV, human papillomavirus; AAV, ANCA-associated vasculitis; PR3, proteinase 3; GCA, giant cell arteritis; PMR, polymyalgia rheumatica; MCTD, mixed connective tissue disease. igg4-RD, IgG4-Related Disease.

**Table 3:** Main stratified screening protocols by disease.

Disease	Stratified Screening Protocol
IIMs	Risk-stratified approach: basic tests for standard risk; CT scans, tumour markers, PET-CT for high-risk (e.g., TIF1γ+).
Sjögren’s Syndrome	Follow general guidelines, monitoring potential warning features (e.g., parotid enlargement).
Systemic Sclerosis	Tailored based on autoantibodies (e.g., ARA+ or ACA/ATA−) and clinical features; standard plus extended study in high-risk patients during first 3–5 years.
SLE	General population guidelines plus annual cervical screening for immunosuppressed women; urine cytology considered post-cyclophosphamide.
Rheumatoid Arthritis	Follow general guidelines; emphasise cervical/skin cancer checks in immunosuppressed; no added screening unless warning features are present.
Antiphospholipid Syndrome	No systematic screening beyond standard population protocols; consider cancer in refractory or atypical cases.
AAV	General population guidelines; focus on skin exams for those exposed to immunosuppressants.
GCA/PMR	Standard guidelines; assess atypical/refractory cases for paraneoplastic syndromes.
Sarcoidosis	General vigilance; screen for haematologic malignancies in high-risk or atypical presentations.
MCTD	No intensified screening recommended; evaluate for malignancy in atypical or treatment-refractory cases.
IgG4-RD	In addition to general population recommendations, cancer screening should be guided by the presence of warning features.
VEXAS Syndrome	Regular monitoring for haematologic malignancy
Eosinophilic Fasciitis	In addition to general population recommendations, cancer screening should be guided by the presence of warning features.

IIMs, idiopathic inflammatory myopathies; CT, computed tomography; PET-CT, positron emission tomography–computed tomography; TIF1γ, transcriptional intermediary factor 1γ; ARA, anti-RNA polymerase III; ACA, anti-centromere antibodies; ATA, anti-topoisomerase I; SLE, systemic lupus erythematosus; AAV, ANCA-associated vasculitis; GCA, giant cell arteritis; PMR, polymyalgia rheumatica; MCTD, mixed connective tissue disease. IgG4-RD, IgG4-related disease.

## Data Availability

Not applicable.
